# Supplementary material for: Cooperative role of PACT and ADAR1 in preventing aberrant PKR activation by self-derived double-stranded RNA
Source: Nat Commun. 2025 Apr 5;16:3246. doi: 10.1038/s41467-025-58412-2 (PMC11971382; doi:10.1038/s41467-025-58412-2)
Supplement: Supplementary file 1 — Supplementary Information [file 41467_2025_58412_MOESM1_ESM.pdf]

Supplementary information for

Cooperative Role of PACT and ADAR1 in Preventing Aberrant PKR Activation by Self-Derived Double-Stranded RNA

Lavanya Manjunath, Gisselle Santiago, Pedro Ortega, Ambrocio Sanchez, Sunwoo Oh, Alexander Garcia, Junyi Li, Dana Duong, Elodie Bournique, Alexis Bouin, Bert L. Semler, Dheva Setiaputra & Rémi Buisson

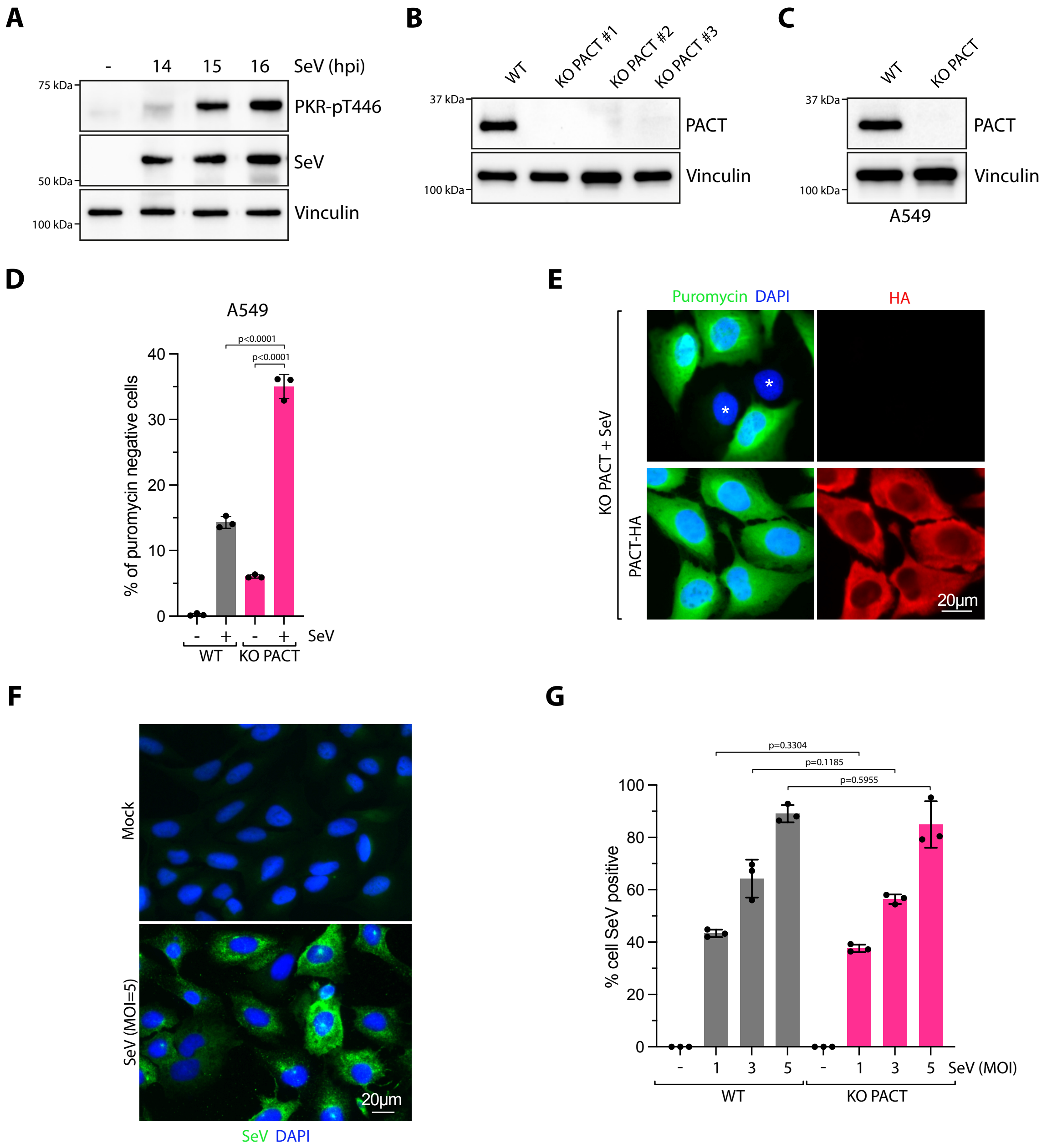

Supplementary Figure 1: A. U2OS WT cells were infected with SeV (MOI=1) and collected at 14 hpi, 15 hpi, and 16 hpi. The levels of PKR phosphorylation (PKR-pT446) and other indicated proteins were monitored by western blot. **B-C.** Analysis of PACT levels by Western blot with indicated antibodies in U2OS (**B**) or A549 (**C**) wildtype or PACT KO cells. **D.** Quantification of puromycin-negative cells in cells infected with SeV-infected A549 WT or PACT KO cells (MOI=1; 24hpi). Mean values ± SD (Number of biological replicates, *n* = 3). *P*-values were calculated by two-way ANOVA. **E.** Representative immunofluorescence for puromycin and HA signal (PACT-HA) in U2OS PACT KO cells or PACT KO cells complemented with PACT-HA infected with SeV (MOI=1, 24hpi). Cells undergoing translation arrest are marked with an asterisk. **F-G.** U2OS WT or PACT KO cells were infected at the indicated MOI by SeV for 24hpi, and SeV-infected cells were detected using RNA-FISH targeting the SeV genome. Representative RNA-FISH images of U2OS cells infected with SeV (MOI=5, 24 hpi) are shown in **F**. The percentage of cells positive for SeV was then quantified in cells positive for RNA-FISH signal (**G**). Mean values ± SD (Number of biological replicates, *n* = 3). *P*-values were calculated by one-way ANOVA. Source data are provided as a Source Data file.

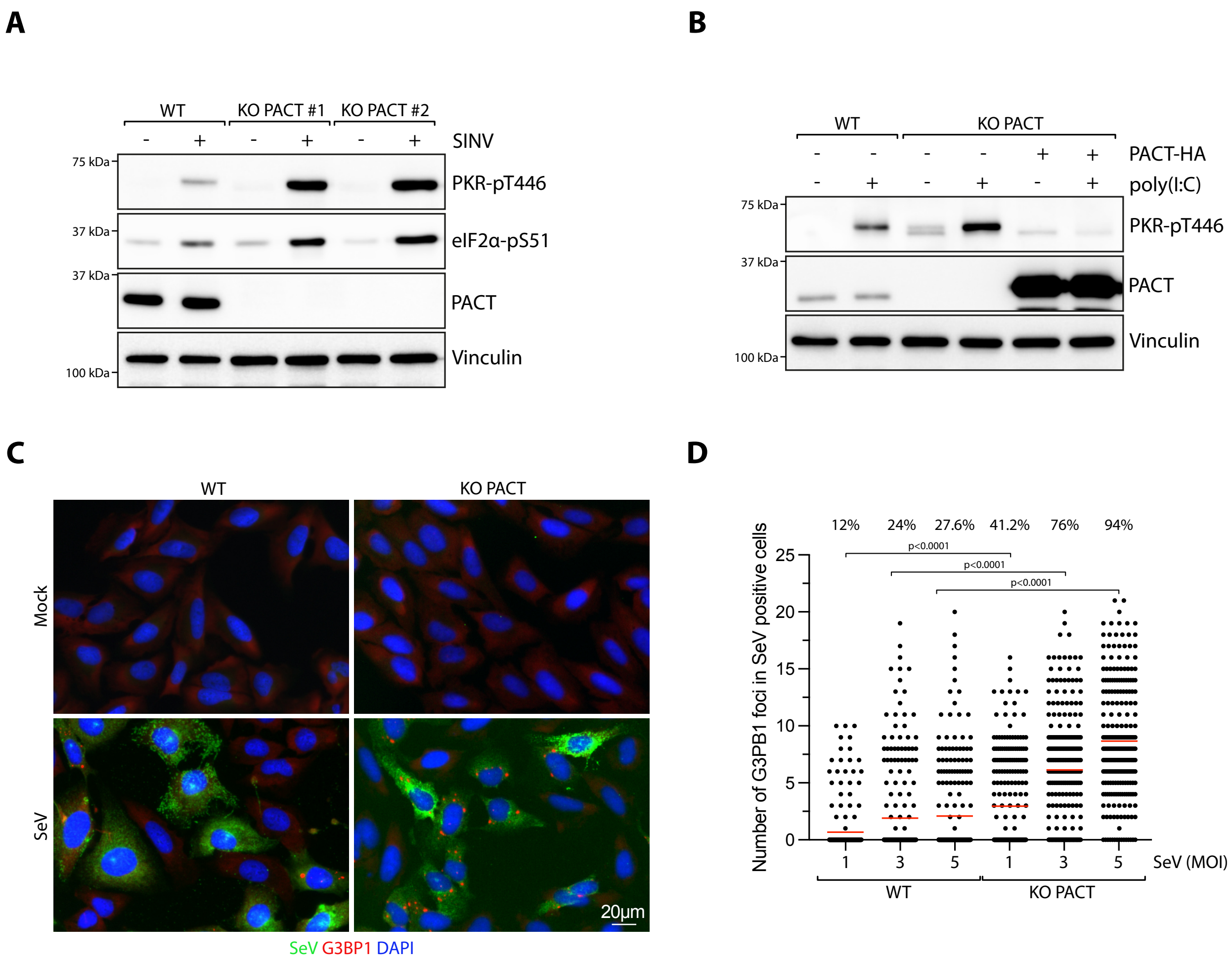

Supplementary Figure 2: A. U2OS WT and PACT KO cells were infected with SINV (MOI=1, 16hpi). The levels of the indicated proteins were monitored by western blot. **B.** U2OS WT and PACT KO cells were transfected with poly(I:C) (200ng/ml; 6h) in the presence of absence of PACT-HA expression. The levels of the indicated proteins were monitored by western blot. **C**. U2OS WT and PACT KO cells were infected with SeV (MOI=1, 24 hpi). G3BP1 was monitored using immunofluorescence, and SeV was assessed via RNA-FISH; representative images are shown. **D.** Quantification of the number of G3BP1 foci in individual cells, as described in Supplementary Figure 2C. Red lines indicate the mean (Number of cells, n = 250). Top: percentage of cells with G3BP1 foci. P-values were calculated using one-way ANOVA. Source data are provided as a Source Data file.

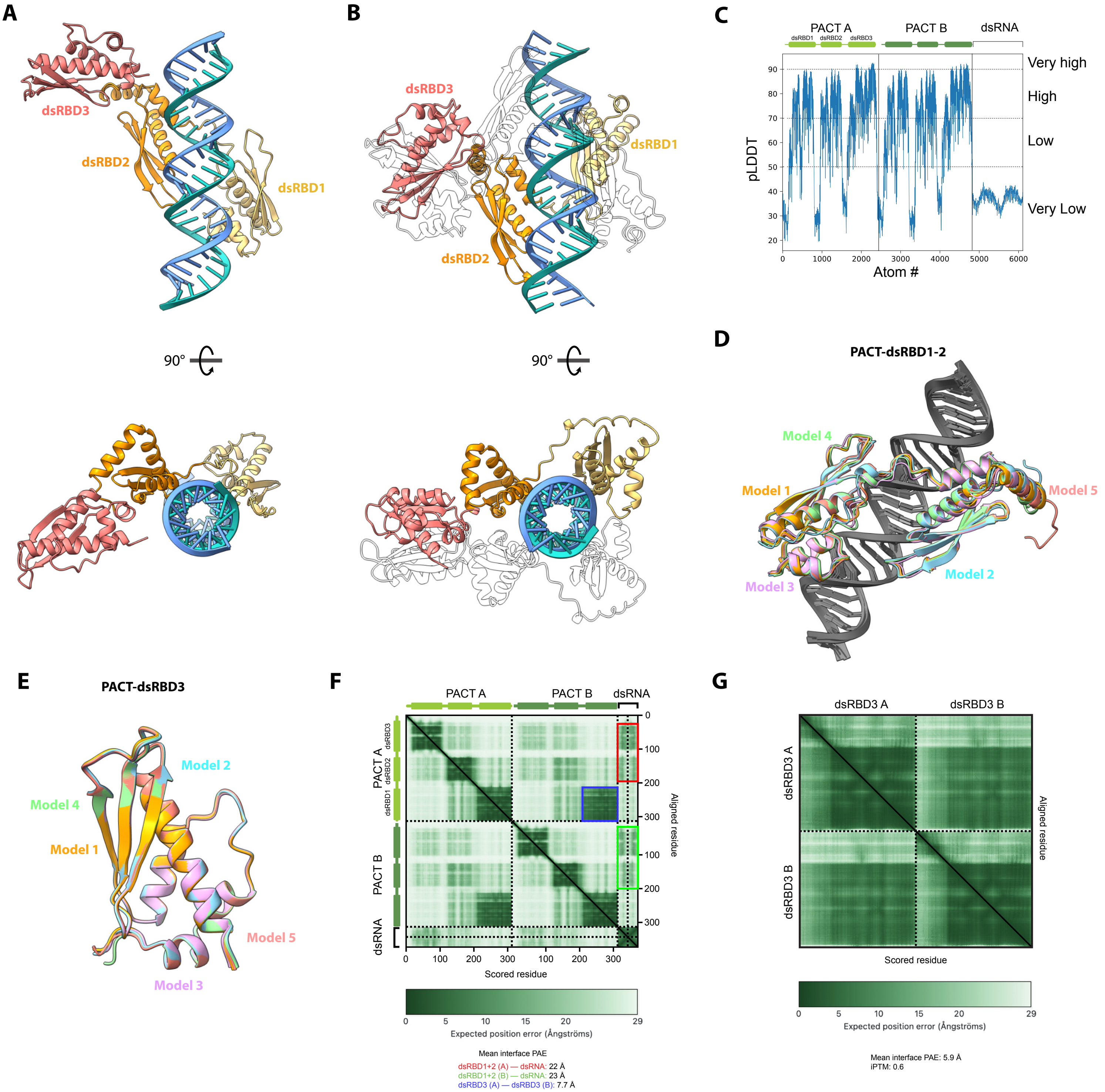

Supplementary Figure 3: A. Top-ranked model predicted by AlphaFold 3 of PACT monomer (residues 21 to 313) binding to dsRNA (blue). Each dsRBD was color-coded as indicated. **B.** Top-ranked model predicted by AlphaFold 3 of PACT dimer (residues 21 to 313) binding to dsRNA (blue). Each dsRBD from one monomeric PACT was color-coded. **C.** pLDDT scores across PACT protein sequence (residues 1–313). **D.** Superposition of five top-ranked models predicted by AlphaFold 3 of monomeric PACT-dsRBD1-2 (residues 21 to 214). **E.** Superposition of five top-ranked models predicted by AlphaFold 3 of PACT-dsRBD3 (residues 211 to 313). **F.** Predicted Aligned Error (PAE) matrix of dimeric PACT (residues 1–313) with dsRNA. The dashed black lines indicate the molecule's boundaries. **G.** Predicted Aligned Error (PAE) matrix of PACT-dsRBD3 (residues 212-313). The dashed black lines indicate the molecule's boundaries.

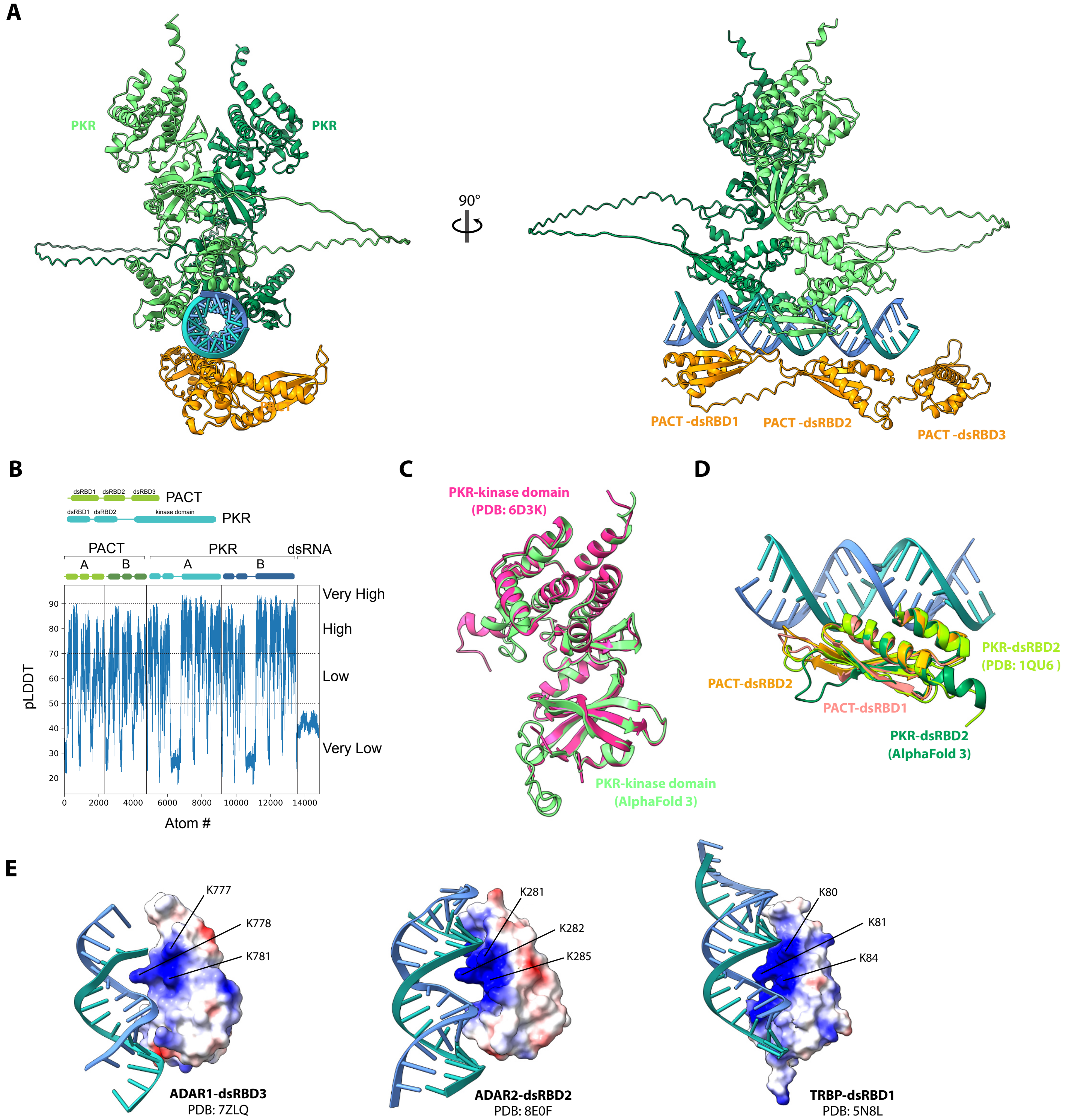

Supplementary Figure 4: A. Top-ranked model predicted by AlphaFold 3 of PKR dimer and PACT monomer (residues 21 to 313) binding to dsRNA (blue). **B.** pLDDT scores across PACT protein sequence (residues 1–313) and PKR protein sequence (residues 1–551). **C.** Superposition of PKR kinase domain (residues 256 to 544) predicted with AlphaFold 3 (green) or determined by X-ray diffraction (pink). **D.** Superposition of PKR dsRBDs (residues 101 to 177) predicted with AlphaFold 3 (dark green) or determined by X-ray diffraction (light green) with PACT-dsRBD1 (red: residues 35 to 99) and PACT-dsRBD2 (orange: residues 127 to 194) predicted with AlphaFold 3. **E.** Electrostatic surface potentials of ADAR1-dsRBD3 (PDB: 7ZLQ), ADAR2-dsRBD2 (PDB: 8E0F), and TRBP-dsRBD1 (PDB: 5N8L) were color-coded: red for negatively charged amino acids, white for neutral residues, and blue for positively charged amino acids. Lysines in close proximity to dsRNA were highlighted.

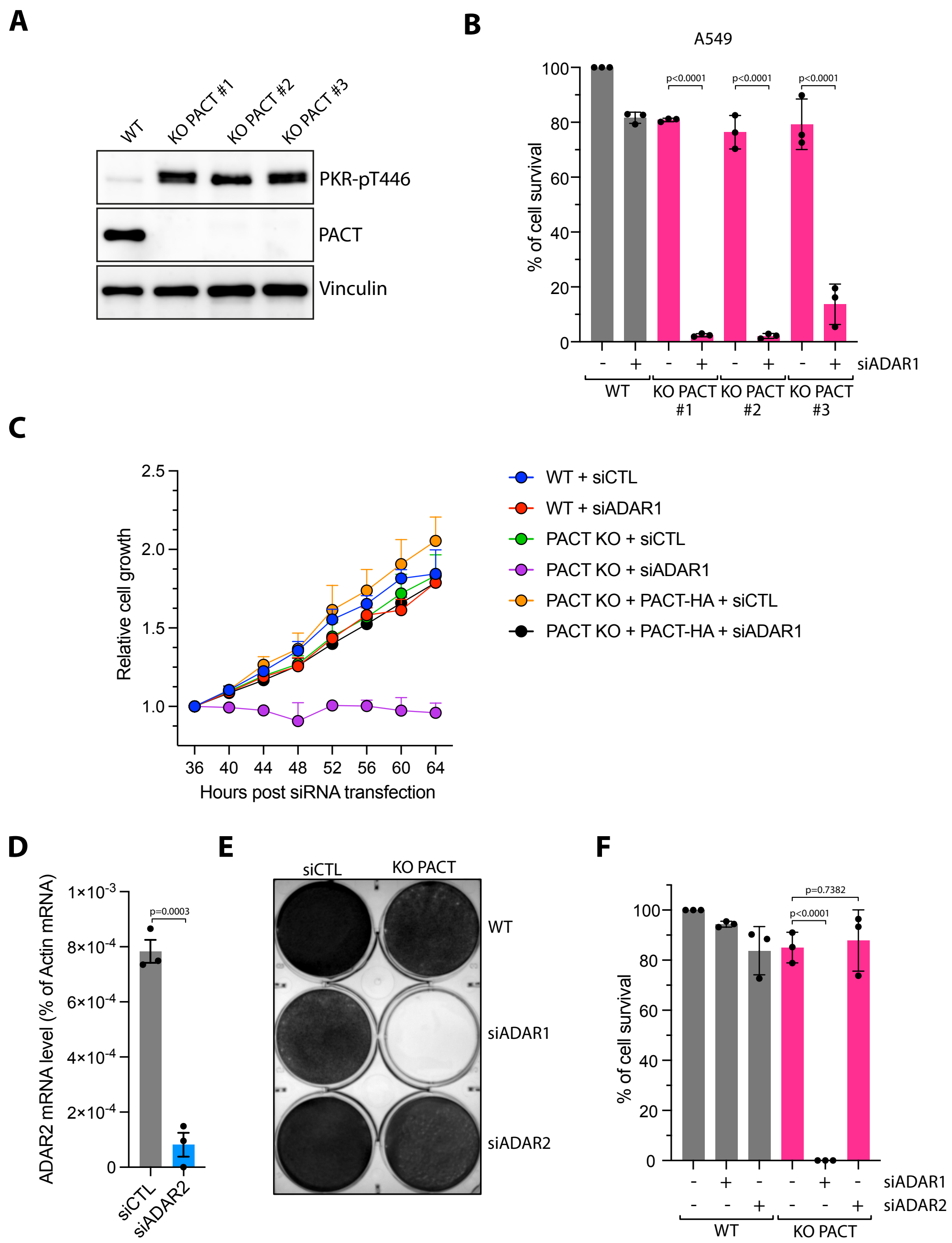

Supplementary Figure 5: A. The levels of PKR-pT446, PACT, and Vinculin were monitored by Western Blot in the U2OS WT or PACT KO cells. **B.** A549 cell lines were knocked down with siCTL or siADAR1. Cell survival were quantified with Alamar blue cell viability assay 6 days following siRNA transfection. Mean values ± SD (Number of biological replicates, *n* = 3). **C.** Indicated U2OS cell lines were transfected with siRNA control (siCTL) or against ADAR1 (siADAR1). Cell growth was then monitored starting at 36h following siRNA transfection. 9 image fields were analyzed per well. Mean values ± SD (Number of biological replicates, *n* = 3). **D.** The levels of ADAR2 mRNA were analyzed by RT-qPCR in U2OS cells transfected with siRNA control (siCTL) or against ADAR2 (siADAR2) for 48h. **E.** Crystal violet staining showing the viability of U2OS WT or PACT KO transfected with siCTL, siADAR1, or siADAR2. Cells were stained with Crystal violet 6 days following transfection with siRNA. **F.** Indicated cell lines were transfected with siCTL, siADAR1, or siADAR2. Cell survival was quantified with Alamar blue cell viability assay 6 days following siRNA transfection. Mean values ± SD (Number of biological replicates, *n* = 3). Source data are provided as a Source Data file.

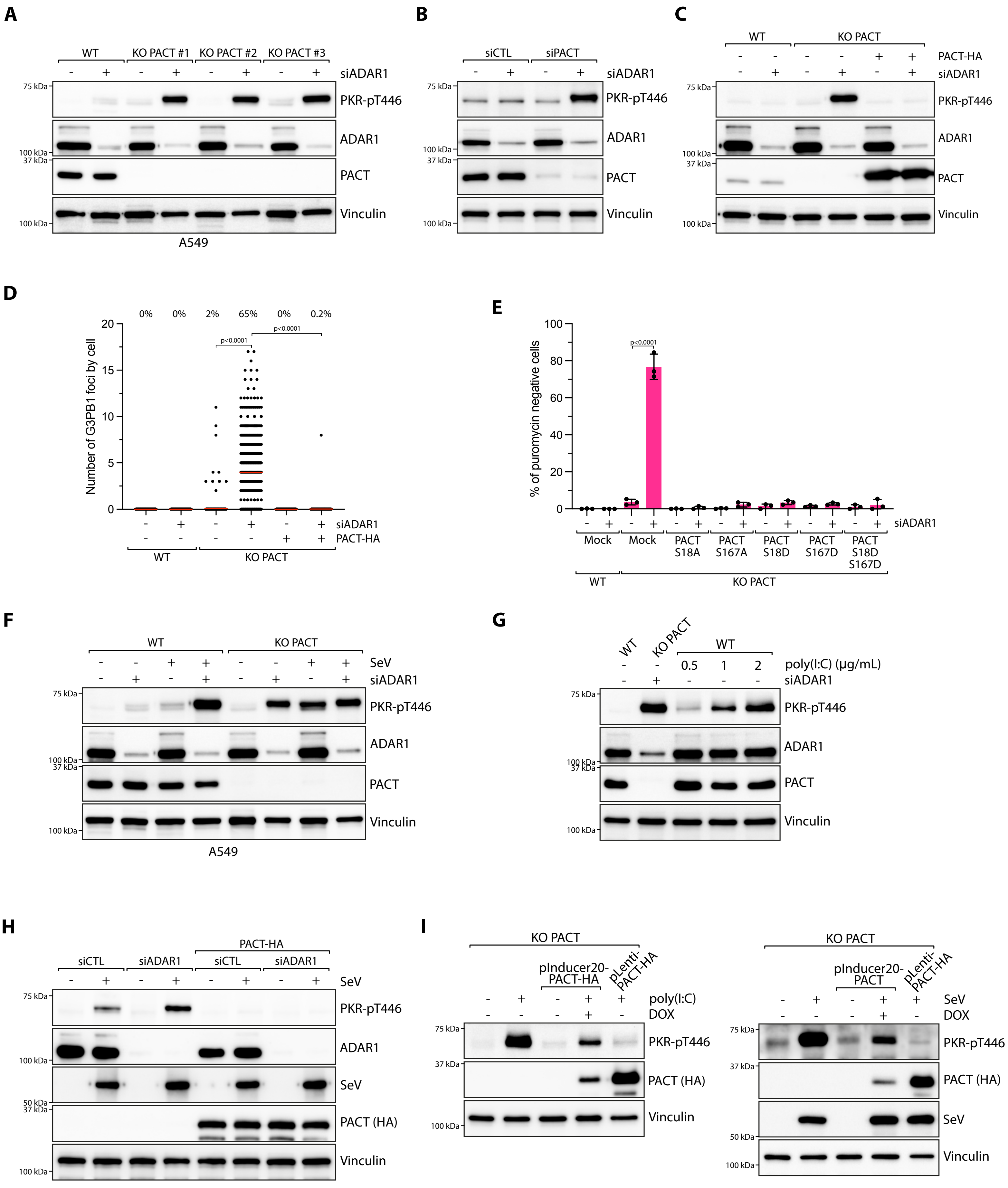
A
WT
KO PACT #1
KO PACT #2
KO PACT #3
siADAR1
75 kDa
100 kDa
37 kDa
PKR-pT446
ADAR1
PACT
Vinculin
A549
B
siCTL
siPACT
siADAR1
PKR-pT446
ADAR1
PACT
Vinculin
C
WT
KO PACT
PACT-HA
siADAR1
PKR-pT446
ADAR1
PACT
Vinculin
D
0%
0%
2%
65%
0%
0.2%
p<0.0001
p<0.0001
Number of G3PB1 foci by cell
siADAR1
PACT-HA
WT
KO PACT
E
% of puromycin negative cells
p<0.0001
siADAR1
Mock
Mock
PACT S18A
PACT S167A
PACT S18D
PACT S167D
PACT S18D S167D
WT
KO PACT
F
WT
KO PACT
SeV
siADAR1
PKR-pT446
ADAR1
PACT
Vinculin
A549
G
WT
KO PACT
WT
poly(I:C) (µg/mL)
siADAR1
0.5
1
2
PKR-pT446
ADAR1
PACT
Vinculin
H
PACT-HA
siCTL
siADAR1
siCTL
siADAR1
SeV
PKR-pT446
ADAR1
SeV
PACT (HA)
Vinculin
50 kDa
I
KO PACT
pInducer20-PACT-HA
pLenti-PACT-HA
poly(I:C)
DOX
PKR-pT446
PACT (HA)
Vinculin
KO PACT
pInducer20-PACT
pLenti-PACT-HA
SeV
DOX
PKR-pT446
PACT (HA)
SeV
Vinculin

Supplementary Figure 6: A. A549 WT and PACT KO cells were knocked down with siRNA control (siCTL) or against ADAR1 (siADAR1) for 48h. The levels of the indicated proteins were monitored by western blot. **B.** U2OS indicated siRNAs were knocked down in U2OS cells. The levels of the indicated proteins were monitored by western blot. **C.** U2OS WT and PACT KO cells were knocked down with siRNA control (siCTL) or against ADAR1 (siADAR1) for 48h. When indicated, PACT-HA was expressed in cells. The levels of the indicated proteins were monitored by western blot. **D.** Quantification of the number of G3BP1 foci by cell in U2OS WT and PACT KO cells knocked down with siRNA control (siCTL) or against ADAR1 (siADAR1) for 48h. When indicated, PACT-HA was expressed in cells. Red lines indicate the mean (Number of cells, $n = 500$). Top; percentage of cells with G3BP1 foci. *P*-values were calculated with a two-tailed *t*-test. **E.** Quantification of puromycin-negative cells in U2OS WT or PACT KO cells expressing the indicated PACT constructs and knocked down with siCTL or siADAR1 for 48h. Mean values ± SD (Number of biological replicates, $n = 3$). P-values were calculated using one tailed t-test. **F.** A549 WT and PACT KO cells were knocked down with siRNA control (siCTL) or against ADAR1 (siADAR1) and subsequently infected with SeV (MOI=1, 24hpi). The levels of PKR phosphorylation (PKR-pT446) and other indicated proteins were monitored by western blot. **G.** U2OS WT were transfected with the indicated concentration of poly(I:C) for 6h. The levels of PKR phosphorylation (PKR-pT446) were monitored by western blot and compared to PKR-pT446 levels of PACT KO cells knocked down with siRNA against ADAR1 (siADAR1). **H.** U2OS WT cells or U2OS cells expressing PACT-HA were knocked down with siRNA control (siCTL) or against ADAR1 (siADAR1) and subsequently infected with SeV (MOI=1, 24hpi). The levels of PKR phosphorylation (PKR-pT446) and other indicated proteins were monitored by western blot. **I**. U2OS PACT KO cells were complemented with PACT WT using two lentiviral systems expressing PACT at different levels, and cells were infected with SeV (MOI=1, 24hpi) or transfected with poly(I:C) (800 ng/mL; 6h). The levels of PKR phosphorylation (PKR-pT446) and other indicated proteins were monitored by western blot. Source data are provided as a Source Data file.

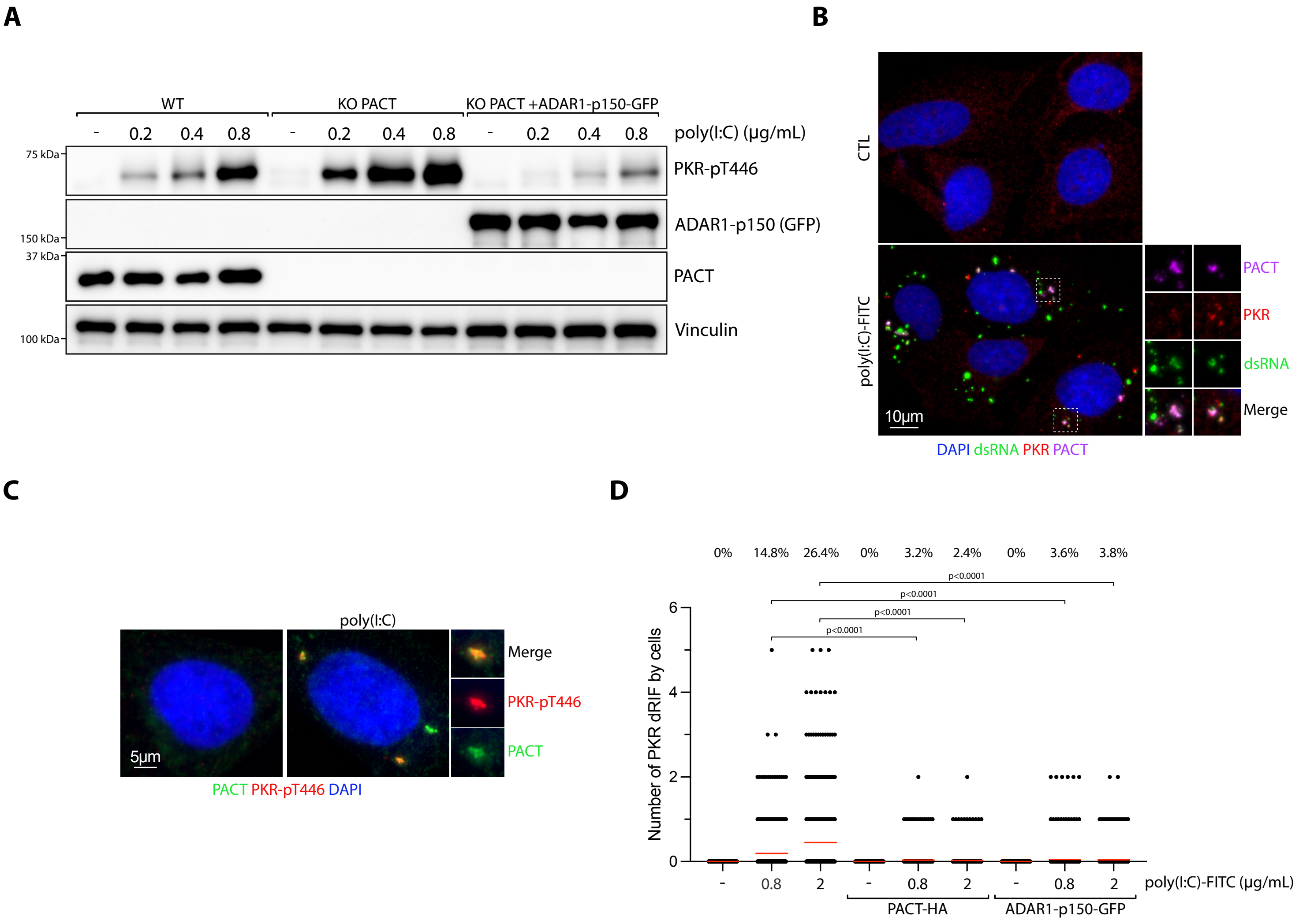

Supplementary Figure 7: A. U2OS WT, PACT KO, or PACT KO + ADAR1-p150-GFP cells were transfected with the indicated concentration of poly(I:C) for 6h. The levels of the indicated proteins were monitored by western blot. **B**. Representative immunofluorescence for PKR, PACT, and dsRNA (poly(I:C)-FITC) localization in U2OS WT cells transfected with poly(I:C)-FITC (800 ng/mL) for 6 h. **C.** Representative immunofluorescence for PKR-pT446 and PACT localization in U2OS WT cells transfected with poly(I:C) (800 ng/mL) for 6 h. **D.** Quantification of the number of PKR foci per cell associated with dRIFs in U2OS WT, U2OS WT + PACT-HA, or U2OS WT + ADAR1-p150-GFP cells transfected with indicated concentration of poly(I:C)-FITC for 6h. (Number of cells, n = 250). Source data are provided as a Source Data file.

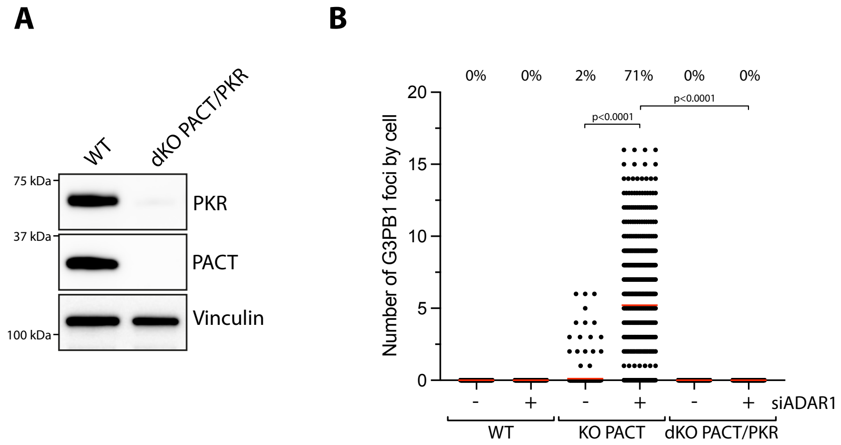

Supplementary Figure 8: A. Levels of PKR, PACT, and Vinculin were monitored by western blot in U2OS WT or PACT/PKR dKO cells. **B.** U2OS WT, PACT KO, or PACT/PKR dKO cells knocked down with siRNA control (siCTL) or against ADAR1 (siADAR1) for 48h, and the number of G3BP1 foci were quantified. Red lines indicate the mean (Number of cells, $n = 500$). Top; percentage of cells with G3BP1 foci. P-values were calculated with a two-tailed t-test. Source data are provided as a Source Data file.
