# Supplementary material for: Cooperative role of PACT and ADAR1 in preventing aberrant PKR activation by self-derived double-stranded RNA
Source: Nat Commun. 2025 Apr 5;16:3246. doi: 10.1038/s41467-025-58412-2 (PMC11971382; doi:10.1038/s41467-025-58412-2)
Supplement: Supplementary file 2 — Reporting Summary [file 41467_2025_58412_MOESM2_ESM.pdf]

nature portfolio

Corresponding author(s): Rémi Buisson

Last updated by author(s): Mar 9, 2025

Reporting Summary

Nature Portfolio wishes to improve the reproducibility of the work that we publish. This form provides structure for consistency and transparency in reporting. For further information on Nature Portfolio policies, see our Editorial Policies and the Editorial Policy Checklist.

Statistics

For all statistical analyses, confirm that the following items are present in the figure legend, table legend, main text, or Methods section.

n/a	Confirmed	
☐	☒	The exact sample size (n) for each experimental group/condition, given as a discrete number and unit of measurement
☐	☒	A statement on whether measurements were taken from distinct samples or whether the same sample was measured repeatedly
☐	☒	The statistical test(s) used AND whether they are one- or two-sided<br>*Only common tests should be described solely by name; describe more complex techniques in the Methods section.*
☒	☐	A description of all covariates tested
☒	☐	A description of any assumptions or corrections, such as tests of normality and adjustment for multiple comparisons
☐	☒	A full description of the statistical parameters including central tendency (e.g. means) or other basic estimates (e.g. regression coefficient) AND variation (e.g. standard deviation) or associated estimates of uncertainty (e.g. confidence intervals)
☐	☒	For null hypothesis testing, the test statistic (e.g. F, t, r) with confidence intervals, effect sizes, degrees of freedom and P value noted<br>*Give P values as exact values whenever suitable.*
☒	☐	For Bayesian analysis, information on the choice of priors and Markov chain Monte Carlo settings
☒	☐	For hierarchical and complex designs, identification of the appropriate level for tests and full reporting of outcomes
☒	☐	Estimates of effect sizes (e.g. Cohen's d, Pearson's r), indicating how they were calculated

Our web collection on statistics for biologists contains articles on many of the points above.

Software and code

Policy information about availability of computer code

Data collection	Gel images were acquired using a Chemidoc MP (BioRad)<br>qRT–PCR was performed in QuantStudio 3 (Applied Biosystems) using Applied Biosystems QuantStudio software.<br>IF image were recorded using a Leica DMi8 THUNDER microscope
Data analysis	Graphs were generated using Prism 9 (GraphPad).<br>IF images were quantify using CellProfiler (https://cellprofiler.org/) version is 4.0.7

For manuscripts utilizing custom algorithms or software that are central to the research but not yet described in published literature, software must be made available to editors and reviewers. We strongly encourage code deposition in a community repository (e.g. GitHub). See the Nature Portfolio guidelines for submitting code & software for further information.

Data

Policy information about availability of data

All manuscripts must include a data availability statement. This statement should provide the following information, where applicable:

- Accession codes, unique identifiers, or web links for publicly available datasets
- A description of any restrictions on data availability
- For clinical datasets or third party data, please ensure that the statement adheres to our policy

The source data underlying Figures 1B, and Supplementary Figure 5B are provided as a supplementary data file. All sequencing data were deposited in NCBI

Sequence Read Archive, using the Bioproject Accession upon publication

Research involving human participants, their data, or biological material

Policy information about studies with human participants or human data. See also policy information about sex, gender (identity/presentation), and sexual orientation and race, ethnicity and racism.

Reporting on sex and gender	not applicable
Reporting on race, ethnicity, or other socially relevant groupings	not applicable
Population characteristics	not applicable
Recruitment	not applicable
Ethics oversight	not applicable

Note that full information on the approval of the study protocol must also be provided in the manuscript.

Field-specific reporting

Please select the one below that is the best fit for your research. If you are not sure, read the appropriate sections before making your selection.

- [x] Life sciences
- [ ] Behavioural & social sciences
- [ ] Ecological, evolutionary & environmental sciences

For a reference copy of the document with all sections, see nature.com/documents/nr-reporting-summary-flat.pdf

Life sciences study design

All studies must disclose on these points even when the disclosure is negative.

Sample size	Sample sizes were chosen according to accepted standards in the field. Sample size was not pre-determined using statistics tolls. As indicated in the figure legends, minimal size of analyzed biological samples was "3". Statistical analysis (as described in respective figure legends) was used to calculate statistical significance of obtained results. The individual p-values are indicated in figures or in figure legends.
Data exclusions	No data were excluded.
Replication	All experiments have been repeated in multiple successfully independent experiments (3 times or more).
Randomization	We had a limited number of biological samples. The analysis was self-normalized to the sample, so randomization of samples would not be a relevant method.
Blinding	As the analysis required comparisons against a known controls and knockdown targets were selected for their likely relevance to the biological pathway, blinding would not provide much reduction of potential bias in the analysis. However, performance and analyses of experiments were independently conducted by co-authors.

Reporting for specific materials, systems and methods

We require information from authors about some types of materials, experimental systems and methods used in many studies. Here, indicate whether each material, system or method listed is relevant to your study. If you are not sure if a list item applies to your research, read the appropriate section before selecting a response.

Materials & experimental systems

n/a	Involved in the study	
[ ]	[x]	Antibodies
[ ]	[x]	Eukaryotic cell lines
[x]	[ ]	Palaeontology and archaeology
[x]	[ ]	Animals and other organisms
[x]	[ ]	Clinical data
[x]	[ ]	Dual use research of concern
[x]	[ ]	Plants

Methods

n/a	Involved in the study	
[x]	[ ]	ChIP-seq
[x]	[ ]	Flow cytometry
[x]	[ ]	MRI-based neuroimaging

Antibodies

Antibodies used	GAPDH Rabbit polyclonal EMD Millipore (#ABS16) 1/20,000 WB<br>STAT1-pY701 Rabbit monoclonal Cell Signaling (#9167) 1/1000 WB<br>G3BP1 Rabbit monoclonal BD Biosciences (#611127) 1/500 IF<br>PKR Mouse monoclonal BD Biosciences (#610764) 1/1000 WB<br>PKR-pT446 Rabbit monoclonal Abcam (ab32036) 1/1000 WB<br>Puromycin Mouse monoclonal EMD Millipore (#MABE343) 1/500 IF<br>Vinculin Mouse monoclonal Sigma (V9264) 1/5000 WB<br>PACT (D9NJ6J) Rabbit monoclonal Cell Signaling (#13490) 1/1000 WB, 1/500 IF<br>HA Rabbit polyclonal Invitrogen #71-5500 1/1000 WB, 1/500 IF<br>ADAR1 Rabbit Monoclonal Cell signaling #81284 1/1000 WB<br>eIF2a-pS51 Rabbit monoclonal Abcam #32157 1/3000 WB<br>Enterovirus (PV-VP1) Mouse monoclonal Dako #M7064 1/500 WB<br>EV-71 VP1 Rabbit monoclonal Abcam (ab308205) 1/1000 WB, 1/500 IF<br>GFP Mouse monoclonal Invitrogen #33-2600 1/1000 IF<br>GFP Rabbit polyclonal Invitrogen #A11122 1/500 IF<br>SeV Rabbit polyclonal MBL #PD029 1/5000 WB
Validation	Antibodies specificity against PKR, PKR-pT446, PACT, and ADAR1 were confirmed in this manuscript using knockout cell or knockdown lines for the corresponding protein target. GAPDH, Vinculin, Puromycin, eIF2a, GFP and HA antibodies were validated by the manufacturer for western blots and referenced in other papers that are included in the manufacturer website. SeV, EV-71 VP1 and PV-VP1 were validated by comparing cell infected to uninfected. STAT1-pY701 and G3PB1 antibodies were validated in previous publication from our laboratory (PMID: 39475651, PMID: 36781883, PMID: 34389714)

Eukaryotic cell lines

Policy information about cell lines and Sex and Gender in Research

Cell line source(s)	U2OS and A549 cell lines were purchased from either ATCC or Sigma-Aldrich. Knockout cell lines were derived from U2OS cell lines as described in the method section.
Authentication	All cell lines were obtained from commercial repositories (ATTC, Sigma Aldrish). Upon receipt, the cell lines were expanded and frozen stocks were created. For the experiments described in this article, cell lines were not continuously kept in culture for more than 3 months. These cell lines were not authenticated
Mycoplasma contamination	All cell lines were tested repetitively for Mycoplasma contamination and all cell lines were tested negative for Mycoplasma
Commonly misidentified lines (See ICLAC register)	No commonly misidentified cell lines were used in the study
