# Supplementary material for: Cooperative role of PACT and ADAR1 in preventing aberrant PKR activation by self-derived double-stranded RNA
Source: Nat Commun. 2025 Apr 5;16:3246. doi: 10.1038/s41467-025-58412-2 (PMC11971382; doi:10.1038/s41467-025-58412-2)
Supplement: Supplementary file 3 — Description of Additional Supplementary Files [file 41467_2025_58412_MOESM3_ESM.pdf]

Description of Additional Supplementary Files

File Name: Supplementary Data 1
Description: The list of genes identified in the CRISPR-Cas9 screens shown in Figures 1b and 5b.

File Name: Supplementary Data 2
Description: The list of reagents, including DNA primers, gRNAs, siRNAs, and antibodies used in this study.

File Name: Supplementary Movie 1
Description: Visualization of the dimeric PACT structure bound to dsRNA, modeled by AlphaFold3.

File Name: Supplementary Movie 2
Description: Visualization of dimeric PKR and monomeric PACT structures bound to dsRNA, modeled by AlphaFold3.
